# Protein Fraction-Dependent Antioxidant Responses to Thermal Processing in Korean Native Black Goat Extracts: A Screening and Multivariate Analysis

**DOI:** 10.3390/foods15101809

**Published:** 2026-05-20

**Authors:** Woo-Young Son, Jun Hwang, Hyun-Wook Kim

**Affiliations:** 1 Division of Animal Bioscience & Integrated Biotechnology, Gyeongsang National University, Jinju 52828, Republic of Korea; sonwy001223@naver.com (W.-Y.S.); hwangjun1116@naver.com (J.H.); 2 Department of GreenBio Science, Gyeongsang National University, Jinju 52725, Republic of Korea

**Keywords:** antioxidant activity, Korean native black goat, meat extract, myofibrillar protein, thermal processing

## Abstract

Korean native black goat is commonly consumed as thermally processed extracts and is widely perceived to exhibit health-promoting properties; however, the contribution of intrinsic muscle proteins to these functional characteristics remains unclear. This study evaluated the effects of thermal processing on the antioxidant activity of myofibrillar and sarcoplasmic protein fractions using a screening-based approach. Protein fractions were extracted and subjected to various thermal conditions (60–121 °C), followed by analyses of extractable protein, α-amino group content, and antioxidant activities, including 2,2-diphenyl-1-picrylhydrazyl (DPPH) radical scavenging, hydroxyl radical scavenging, iron-chelating ability, and ferric reducing antioxidant power (FRAP). Thermal processing increased α-amino group content, particularly in the myofibrillar fraction, indicating enhanced protein degradation. Antioxidant activities improved with increasing temperature, with myofibrillar proteins showing stronger activity than sarcoplasmic proteins. Sodium dodecyl sulfate–polyacrylamide gel electrophoresis (SDS–PAGE) indicated fragmentation in myofibrillar proteins and aggregation in sarcoplasmic proteins. Cluster analysis supported fraction-dependent differences in antioxidant responses. These findings suggest that thermal processing enhances antioxidant activity in a protein fraction-dependent manner and provide practical insights for optimizing processing conditions of Korean native black goat extracts.

## 1. Introduction

Korean native black goat (*Capra hircus coreanae*) is traditionally consumed in Korea predominantly in the form of thermally processed extracts rather than as fresh meat, reflecting a long-standing dietary practice associated with perceived health-promoting benefits [1]. Despite its cultural and nutritional relevance, the scientific basis underlying the functional properties of Korean native black goat extracts remains insufficiently established, particularly with respect to processing-induced variability. In conventional preparations, prolonged heating, often combined with herbal ingredients, results in complex matrices that obscure the intrinsic contribution of muscle-derived components to the observed bioactivity. Previously, several physiological benefits such as antioxidant and anti-inflammatory activities have been reported in Korean native black goat products, and have frequently been attributed to low-molecular-weight compounds generated during processing [2]. However, most of the studies have considered whole muscle systems or multi-component extracts, limiting mechanistic interpretation due to the coexistence of diverse compounds and uncontrolled interactions. As a result, it remains unclear whether the observed functional properties originate from specific protein fractions or from combined matrix effects.

Thermal processing plays a critical role in changing meat protein functionality by inducing heat-induced denaturation, aggregation, and fragmentation, which can induce structural modification, fragmentation, and exposure of reactive amino acid residues [3]. In meat extract systems, prolonged thermal treatment has been reported to induce partial protein degradation and peptide release even in the absence of exogenous enzymatic hydrolysis [4]. However, the extent of such heat-induced peptide generation is likely to depend strongly on protein structural characteristics and processing conditions, particularly temperature and heating duration [5,6]. Muscle proteins are classified into myofibrillar (salt-soluble) and sarcoplasmic (water-soluble) fractions based on solubility, accounting for approximately 50–60% and 30–35% of total muscle protein, respectively [7]. Protein structure is associated with susceptibility to thermal modification, and thus protein fractionation provides a critical framework for understanding structure–function relationships during meat processing. Myofibrillar proteins are structurally stabilized by intermolecular interactions, whereas sarcoplasmic proteins are more soluble and less structurally constrained [7,8]. These intrinsic differences suggest that the two fractions may follow distinct pathways of thermal-induced structural transformation, leading to differential patterns of peptide generation and functional activity. Nevertheless, fraction-specific responses to thermal processing have not been systematically investigated in meat extract systems.

Despite these limitations, it remains unclear which protein fractions primarily contribute to the antioxidant responses of Korean native black goat extracts and how thermal processing conditions influence their properties. To address this gap, a fraction-based approach was employed to compare the antioxidant responses of myofibrillar and sarcoplasmic proteins under controlled heating conditions. Previous studies have reported that goat meat exhibits distinct oxidative and biochemical characteristics compared with conventional livestock species such as beef and pork, which may influence protein structural responses and redox-related behavior during thermal processing [9,10]. Therefore, the present study was designed as a screening-level investigation to evaluate thermal processing-induced changes in the physicochemical properties and antioxidant activities of myofibrillar and sarcoplasmic protein fractions from Korean native black goat. In addition, multivariate analysis was applied to explore relationships among thermal conditions, protein fractions, and antioxidant response patterns. Such an approach may provide useful preliminary information for optimizing thermal processing conditions in goat meat extract systems.

## 2. Materials and Methods

### 2.1. Raw Materials

Korean native black goats (*Capra hircus coreanae*) used in this study were obtained from a commercial goat farm. A total of six 14-month-old castrated males (average live weight: approximately 45 kg) were slaughtered at a commercial abattoir under standard industrial conditions. At 48 h postmortem, *M. longissimus thoracis et lumborum* muscles were manually separated, vacuum-packaged, and transported to the laboratory under refrigerated conditions (4 °C). Upon arrival, visible subcutaneous fat and excessive connective tissue were carefully removed using sterile knives. The trimmed muscle samples were then used for subsequent protein extraction and stored at 4 °C prior to analysis.

### 2.2. Protein Extraction

Myofibrillar proteins were extracted according to the method of Wu et al. [11] with minor modifications. Trimmed muscle samples were minced and homogenized with four volumes (*v*/*w*) of extraction buffer (10 mM sodium phosphate, pH 7.0, containing 0.1 M NaCl, 2 mM MgCl_2_, and 1 mM EGTA) using a homogenizer at 10,000 rpm for 90 s. The homogenate was centrifuged at 3000× *g* for 15 min at 4 °C, and the supernatant was discarded. The pellet was resuspended in the same buffer, and the washing step was repeated twice to remove sarcoplasmic proteins and soluble components. Following the final wash, the pellet was resuspended in 0.1 M NaCl at a ratio of four volumes relative to the initial sample weight and homogenized for 90 s. The pH of the suspension was adjusted to 6.2 using 1 N HCl under continuous stirring, followed by centrifugation at 3000× *g* for 15 min at 4 °C. This procedure was repeated once more, and the resulting pellet was collected as the myofibrillar protein fraction.

Sarcoplasmic proteins were extracted based on the method of Marino et al. [12] with slight modifications. Muscle samples were homogenized with three volumes (*v*/*w*) of 0.003 M sodium phosphate buffer (pH 7.0) for 3 min using a homogenizer, followed by centrifugation at 8000× *g* for 20 min at 4 °C. The supernatant was filtered through glass wool to remove residual particulates and connective tissue debris and collected as the sarcoplasmic protein fraction. The protein concentration of each fraction was determined using BCA assay (Pierce™ BCA Protein Assay Kits, 23225#, Thermo Fisher Scientific, Waltham, MA, USA). Subsequently, both myofibrillar and sarcoplasmic protein fractions were adjusted to a final concentration of 10 mg/mL using the corresponding buffer solutions prior to thermal treatment to ensure comparable protein availability and minimize variability associated with concentration-dependent aggregation.

### 2.3. Thermal Treatment

Thermal treatments were applied to evaluate the effects of heating conditions on protein structural modification and antioxidant properties. The separated protein solutions (10 mg/mL) were aliquoted into separate tubes corresponding to each thermal condition, with three technical replicates prepared for each protein fraction. A total of six thermal conditions were applied, including 60 °C for 6, 24, and 48 h; 80 °C for 6 h; 100 °C for 6 h; and 121 °C for 6 h. The extended treatment times at 60 °C were designed to reflect mild thermal extraction conditions commonly used in traditional meat extract processing, where extraction is often performed for more than 12 h at relatively low temperatures. In contrast, higher-temperature processing conditions (>100 °C) are generally conducted for shorter periods, typically around 6 h, due to rapid protein denaturation and aggregation under intense heating conditions. Consequently, the experimental design was established based on practical industrial processing conditions while also considering experimental efficiency and the distinct thermal responses expected at different temperature ranges. To reflect such practices, thermal treatments at 60 °C, 80 °C, and 100 °C were conducted using a temperature-controlled water bath (JSIB-22T, JS Research Inc., Gongju, Republic of Korea), whereas the 121 °C treatment was performed using a high-pressure steam sterilizer (autoclave). Following thermal processing, samples were immediately cooled in an ice bath to terminate further reactions and centrifuged at 3000× *g* for 10 min at 4 °C to remove insoluble aggregates formed during heating. The resulting supernatants were collected as thermally treated soluble protein extracts. The supernatants were frozen at −80 °C and lyophilized using a freeze dryer, and the resulting powders were stored at −20 °C until further analysis.

### 2.4. Analysis of Hydrolytic Characteristics

#### 2.4.1. α-Amino Group Content

The content of α-amino groups, representing low-molecular-weight peptides and free amino acids, was determined using the *o*-phthaldialdehyde (OPA) method according to Liu et al. [13] with slight modifications. Briefly, 0.1 mL of sample was mixed with 2 mL of freshly prepared OPA reagent, which consisted of 40 mg/mL of OPA dissolved in a mixture containing 0.1 M sodium tetraborate (50 mL), 20% (*w*/*v*) sodium dodecyl sulfate (5 mL), β-mercaptoethanol (200 μL), and deionized water (42.8 mL). The reaction mixture was incubated at room temperature for 2 min, and the absorbance was measured at 340 nm using a spectrophotometer. A standard curve was prepared using casein as a reference protein, and the α-amino acid content was calculated based on the corresponding absorbance values. The results are expressed as mg casein equivalents per mL.

#### 2.4.2. Protein Electrophoresis

Protein profiles were analyzed by sodium dodecyl sulfate–polyacrylamide gel electrophoresis (SDS–PAGE) according to the method of Laemmli [14]. Samples were diluted to a final concentration of 0.8 mg/mL and mixed with 5× Laemmli sample buffer (Elpis, Daejeon, Republic of Korea) at a ratio of 4:1 (*v*/*v*). The mixtures were heated at 100 °C for 5 min to denature proteins and then cooled to room temperature. Aliquots of 25 μL were loaded onto a polyacrylamide gel consisting of a 4% stacking gel and a 15% separating gel, and electrophoresis was performed using a Mini-PROTEAN Tetra Cell system (Bio-Rad, Hercules, CA, USA). Proteins were first resolved through the stacking gel at 70 V for 10 min, followed by separation in the resolving gel at 100 V for 90 min. After electrophoresis, gels were stained with Coomassie Brilliant Blue R-250 staining solution (0.25% (*w*/*v*) Coomassie Brilliant Blue R-250 (Sigma, St Louis, MO, USA), 50% (*v*/*v*) methanol, 10% (*v*/*v*) acetic acid, and 40% (*v*/*v*) distilled water) and subsequently destained using a solution containing 50% (*v*/*v*) methanol, 10% (*v*/*v*) acetic acid, and 40% (*v*/*v*) distilled water until clear protein bands were visible. Molecular weights of protein bands were estimated using a pre-stained protein marker (Dokdo-mark EBM-1032, Elpis, Daejeon, Republic of Korea).

### 2.5. In Vitro Antioxidant Activity Screening

#### 2.5.1. DPPH Radical Scavenging Activity

DPPH radical scavenging activity was determined according to the method of Nicklisch et al. [15] with minor modifications. Samples were diluted to final concentrations of 0.25, 0.5, 0.75, and 1.0 mg/mL using 0.1 M citrate–phosphate buffer (pH 5.5) containing 0.3% (*v*/*v*) Triton X-100. An aliquot of 1235 μL of each sample solution was mixed with 65 μL of 2 mM DPPH solution prepared in 99.5% methanol. The mixture was incubated in the dark at room temperature for 60 min, and the absorbance was measured at 515 nm using a spectrophotometer. The control was prepared by replacing the sample with methanol, while the blank was prepared by replacing the DPPH solution with methanol. The DPPH radical scavenging activity was calculated as follows: DPPH scavenging activity (%) = [1 − (A_sample_ − A_blank_)/A_control_] × 100.

#### 2.5.2. Hydroxyl Radical Scavenging Activity

Hydroxyl radical scavenging activity was determined based on the method of You et al. [16] with slight modifications. The reaction mixture consisted of 600 μL of 5 mM 1,10-phenanthroline, 600 μL of 5 mM FeSO_4_, and 600 μL of 15 mM EDTA, which were combined with 400 μL of 0.2 M sodium phosphate buffer (pH 7.4). Amounts of 600 μL of sample solution and 800 μL of 0.01% (*v*/*v*) hydrogen peroxide were added to the reaction mixture (2.2 mL), followed by incubation at 37 °C for 60 min. The absorbance was then measured at 536 nm. The control was prepared by replacing the sample with distilled water, while the blank was prepared by replacing hydrogen peroxide with distilled water. Hydroxyl radical scavenging activity was calculated using the following equation: Hydroxyl radical scavenging activity (%) = Hydroxyl radical scavenging activity (%) = [(A_sample_ − A_control_)/(A_blank_ − A_control_)] × 100.

#### 2.5.3. Ferrous Ion Chelating Activity

Ferrous ion (Fe^2+^) chelating activity was measured according to the method of Zhu et al. [17] with slight modifications. Briefly, 1 mL of sample solution was mixed with 0.05 mL of 2 mM FeCl_2_ and incubated at room temperature for 10 min. Subsequently, 0.2 mL of 5 mM ferrozine solution was added, and the mixture was allowed to react for an additional 10 min. The absorbance was measured at 562 nm using a spectrophotometer. The control was prepared by replacing the sample with distilled water. The chelating activity was calculated using the following equation: Ferrous ion chelating activity (%) = [(A_control_ − A_sample_)/A_control_] × 100.

#### 2.5.4. Ferric Reducing Antioxidant Power (FRAP)

Ferric reducing antioxidant power (FRAP) was measured following a modified version of the standard FRAP assay [18]. The FRAP reagent was freshly prepared by mixing 0.3 M acetate buffer (pH 3.6), 20 mM ferric chloride hexahydrate (FeCl_3_·6H_2_O), and 10 mM 2,4,6-tripyridyl-s-triazine (TPTZ) solution in a ratio of 10:1:1 (*v*/*v*/*v*). The reagent was incubated at 37 °C for 30 min prior to use. For the assay, 0.1 mL of sample was mixed with 0.3 mL of distilled water and 3 mL of FRAP reagent. The reaction mixture was incubated at 37 °C in the dark for 30 min, and the absorbance was measured at 593 nm. A standard curve was constructed using FeSO_4_·7H_2_O at concentrations ranging from 200 to 1000 μM. The FRAP values of samples were calculated from the standard curve and are expressed as μM Fe^2+^ equivalents.

#### 2.5.5. Estimation of EC_50_ Values by Linear Interpolation

The half-maximal effective concentration (EC50) values were estimated using a linear interpolation method based on the concentration–response data. A four-parameter logistic (4PL) model was initially considered; however, reliable curve fitting was not achievable, due to instability in absorbance values at higher sample concentrations, which was associated with sample turbidity and suspension effects. In particular, at concentrations required to achieve more than 50% antioxidant activity, absorbance values frequently exceeded 2.0 due to heat-induced protein aggregation and insoluble particle formation, resulting in substantial optical interference. Consequently, sigmoidal concentration–response curves could not be stably fitted using the 4PL model. Therefore, EC50 values were calculated by linear interpolation between the two concentrations immediately above and below 50% activity.

### 2.6. Statistical Analysis

All experiments were performed in triplicate (*n* = 3), and the results are expressed as mean ± standard deviation. Statistical analyses were conducted using SPSS ver. 18.0, IBM Corp., Chicago, IL, USA. Differences among treatment groups, including EC_50_ values, were evaluated by one way analysis of variance (ANOVA), followed by Duncan’s multiple range test to determine significant differences between means. Statistical significance was set at *p* < 0.05. To explore the relationships among samples based on their antioxidant properties, hierarchical cluster analysis was performed. Cluster analysis is an unsupervised statistical method that groups samples based on similarity or distance, allowing the identification of natural patterns within the dataset. In this study, clustering was conducted using Euclidean distance and Ward’s method. Prior to clustering, the data were normalized using z-score normalization to minimize the influence of scale differences among variables. The clustering results are visualized using a dendrogram, which represents the hierarchical relationships among samples and illustrates how clusters are formed at different similarity levels.

## 3. Results and Discussion

### 3.1. Hydrolytic Characteristics

#### 3.1.1. α-Amino Group Content

The α-amino group content, used as an indicator of protein degradation, was significantly affected by protein fraction and thermal processing conditions (*p* < 0.001; Table 1). In the myofibrillar fraction, α-amino group content increased markedly with increasing heating time and temperature, showing a gradual rise at 60 °C followed by a remarkable increase at ≥80 °C. In contrast, the sarcoplasmic fraction exhibited relatively minor changes under mild conditions and showed a significant increase only under more intensive thermal treatments. These results indicate that thermal processing induces protein degradation in a fraction-dependent manner. Myofibrillar proteins, which possess highly ordered structures, initially resist thermal denaturation but become increasingly susceptible to degradation following structural unfolding. This result is consistent with previous studies reporting progressive destabilization of myosin (40–60 °C) and actin (70–80 °C), leading to enhanced breakdown under elevated thermal conditions [19]. Sarcoplasmic proteins, which are inherently soluble and structurally less organized, appear to undergo structural denaturation and aggregation rather than extensive degradation. Similar trends have been reported in thermally processed meat systems, where protein degradation and solubility are strongly influenced by structural organization and heating intensity [5,20]. Thus, these results demonstrate that both protein fraction and thermal processing intensity are critical factors affecting protein degradation, with myofibrillar proteins showing greater susceptibility to heat-induced structural modification under the evaluated conditions. Also, the increase in α-amino group content should be interpreted as reflecting overall protein degradation rather than direct evidence of bioactive peptide formation, as no peptide identification was performed in this study.

#### 3.1.2. Protein Electrophoresis

**Figure 1:**
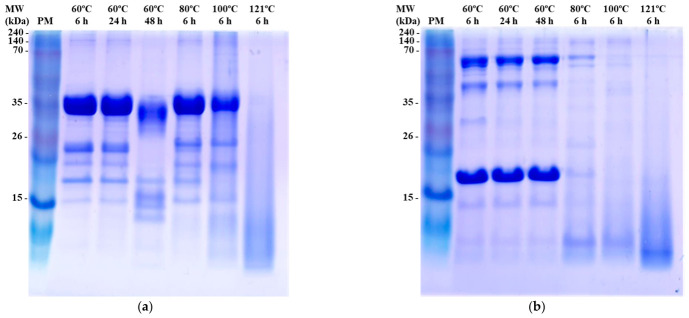
SDS–PAGE profiles of myofibrillar (**a**) and sarcoplasmic (**b**) proteins under different thermal conditions. PM, standard protein marker.

SDS–PAGE analysis further supported the fraction-dependent structural modifications induced by thermal processing (Figure 1). In the myofibrillar fraction (Figure 1a), major high-molecular-weight protein bands, including myosin heavy chain (~200 kDa) and actin (~42 kDa), progressively decreased in intensity with increasing temperature. The concomitant appearance of lower-molecular-weight bands was observed, indicating thermally induced fragmentation of structurally organized proteins. These changes are consistent with the known thermal transitions of myofibrillar proteins, where myosin denatures at approximately 40–60 °C followed by actin at higher temperatures (70–80 °C), resulting in progressive destabilization of the myofibrillar structure [20,21]. In contrast, the sarcoplasmic fraction (Figure 1b) exhibited relatively stable band patterns under mild thermal conditions (60 °C), whereas higher temperatures led to reduced band intensity and increased smearing. This pattern suggests that thermal processing primarily induces structural disruption and aggregation in sarcoplasmic proteins rather than extensive fragmentation [22,23].

When interpreted in conjunction with the α-amino group content results (Table 1), the SDS–PAGE profiles indicate that fragmentation is the dominant process in the myofibrillar fraction, whereas aggregation and structural rearrangement are more prominent in the sarcoplasmic fraction. Similar fraction-dependent protein degradation patterns have been reported in thermally processed meat systems [24]. These findings indicate that thermally induced protein degradation differs substantially between protein fractions, which may subsequently influence their functional properties. In particular, the enhanced fragmentation observed in the myofibrillar fraction provides a structural basis for increased thermally induced protein degradation patterns, which is closely associated with antioxidant activity as discussed in the following section. Taken together, α-amino group content and SDS–PAGE results indicate that thermal processing promotes distinct degradation pathways depending on protein fraction, with fragmentation dominating in myofibrillar proteins and aggregation prevailing in sarcoplasmic proteins. Additional physicochemical analyses, such as pH and turbidity measurements, as well as advanced molecular characterization including LC–MS/MS peptide profiling, are necessary to further elucidate heat-induced structural modifications and the characteristics of potentially bioactive peptides. These analyses should be considered in future studies to provide deeper mechanistic insight into the relationship between protein structural changes and antioxidant activity.

### 3.2. Antioxidant Capacity

#### 3.2.1. Radical Scavenging Activity

**Figure 2:**
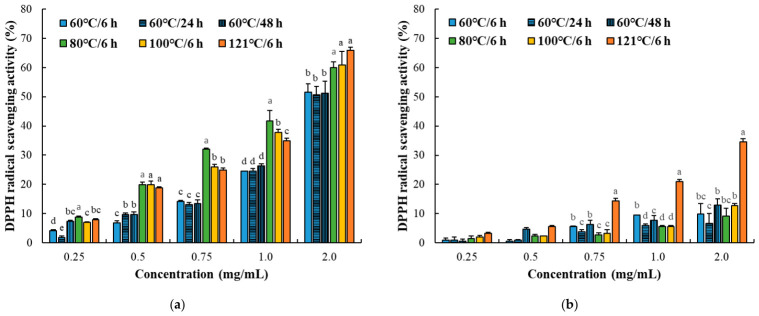
DPPH radical scavenging activity of myofibrillar (**a**) and sarcoplasmic (**b**) protein fractions (0.25–2.0 mg/mL) from Korean native black goat under different thermal conditions. Error bars represent standard deviation (*n* = 3). a–e: Different letters indicate significant differences among treatments within the same concentration (*p* < 0.05).

**Figure 3:**
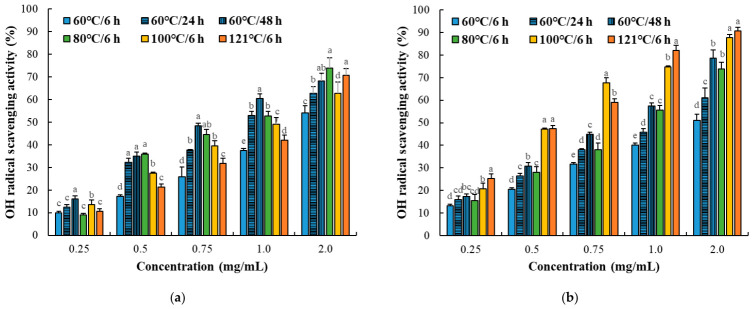
Hydroxyl (OH) radical scavenging activity of myofibrillar (**a**) and sarcoplasmic (**b**) protein fractions (0.25–2.0 mg/mL) from Korean native black goat under different thermal conditions. Error bars represent standard deviation (*n* = 3). a–e: Different letters indicate significant differences among treatments within the same concentration (*p* < 0.05).

DPPH and hydroxyl radical scavenging activities were significantly affected by protein fraction, thermal processing conditions, and sample concentration (Figure 2 and Figure 3; Table 2). In both assays, radical scavenging activity increased in a concentration-dependent manner; however, the magnitude and pattern of response differed markedly between protein fractions and assay types.

In the myofibrillar fraction, radical scavenging activity increased consistently with thermal intensity, with pronounced enhancement observed at ≥80 °C. This trend was supported by lower EC_50_ values compared to the sarcoplasmic fraction, indicating superior antioxidant efficiency. The enhanced activity closely corresponded to increased α-amino group content and fragmentation patterns observed in SDS–PAGE, suggesting that DPPH scavenging activity may be associated with heat-induced structural modification and increased accessibility of electron-donating residues. Moreover, low-molecular-weight peptides and exposed reactive amino acid residues are known to act as effective radical scavengers via hydrogen donation and electron transfer mechanisms [25,26]. In contrast, the sarcoplasmic fraction exhibited relatively moderate and less consistent changes across thermal treatments. This result is consistent with SDS–PAGE results, indicating aggregation-dominant structural modifications rather than extensive fragmentation, which may limit peptide availability and reduce antioxidant efficiency. Notably, DPPH and hydroxyl radical scavenging activities showed distinct response patterns. While DPPH scavenging was strongly influenced by thermal-induced challenge, hydroxyl radical scavenging exhibited greater variability, particularly in the sarcoplasmic fraction. This difference reflects the distinct mechanisms of the assays: DPPH scavenging primarily depends on electron-donating capacity, whereas hydroxyl radical scavenging involves additional processes such as metal ion chelation and inhibition of Fenton-type reactions [27].

Previous studies have similarly reported that antioxidant activities in meat-derived peptide systems are highly assay-dependent and influenced by peptide composition, molecular structure, and processing-induced conformational changes [28,29]. In particular, thermally induced unfolding and fragmentation may enhance exposure of electron-donating amino acid residues, thereby improving DPPH radical scavenging activity, whereas excessive aggregation may reduce accessibility of metal-chelating sites and alter hydroxyl radical scavenging behavior [30]. Consequently, structural factors, including protein aggregation and the accessibility of reactive residues, play a more significant role in hydroxyl radical scavenging. Overall, these results demonstrate that radical scavenging activity in thermally processed protein systems is both fraction-dependent and assay-dependent, suggesting that heat-induced structural modification, protein degradation patterns, and accessibility of reactive residues may differentially influence individual antioxidant responses.

#### 3.2.2. Ferrous Iron Chelating Activity

**Figure 4:**
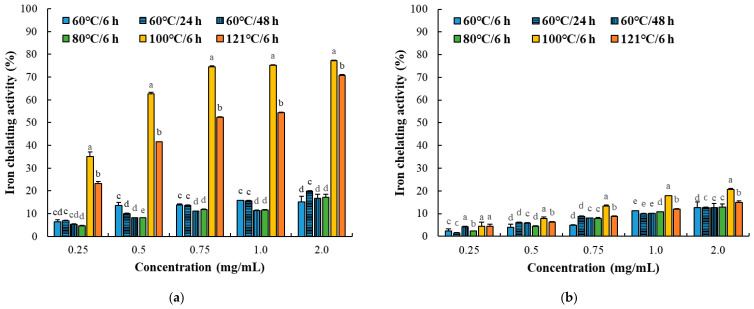
Iron chelating activity of myofibrillar (**a**) and sarcoplasmic (**b**) protein fractions (0.25–2.0 mg/mL) from Korean native black goat under different thermal conditions. Error bars represent standard deviation (*n* = 3). a–e: Different letters indicate significant differences among treatments within the same concentration (*p* < 0.05).

Ferrous iron chelating activity exhibited a distinct response pattern compared to radical scavenging assays (Figure 4; Table 2). In the myofibrillar fraction, EC_50_ values markedly decreased at higher temperatures, particularly at 100 °C and 121 °C (0.39–0.70 mg/mL), indicating a substantial increase in metal-chelating capacity. In contrast, the sarcoplasmic fraction showed generally higher EC_50_ values (2.98–7.50 mg/mL), suggesting lower chelating activity and a weaker response to thermal processing. Unlike radical scavenging activity, which showed a clear association with increasing α-amino group content, iron-chelating activity did not follow the same trend, suggesting that different factors may contribute to this activity. Metal-chelating capacity is generally associated with the availability of functional groups capable of interacting with metal ions [31,32,33]. Recent studies have reported that heat-induced structural modification of meat proteins may alter metal-binding behavior through unfolding, aggregation, and exposure of previously buried reactive sites [29,30]. In addition, low-molecular-weight peptides derived from thermally processed or hydrolyzed meat systems have been reported to exhibit strong iron-chelating activity depending on peptide sequence, amino acid composition, and molecular conformation [28,34]. Thus, the observed improvement in chelating activity at elevated temperatures may be attributed to heat-induced structural changes that increase the accessibility of such binding sites. These results indicate that thermal processing influences antioxidant activity through multiple mechanisms, and that iron-chelating activity is affected by structural factors distinct from those affecting radical scavenging.

#### 3.2.3. Ferric Reducing Antioxidant Power (FRAP)

**Figure 5:**
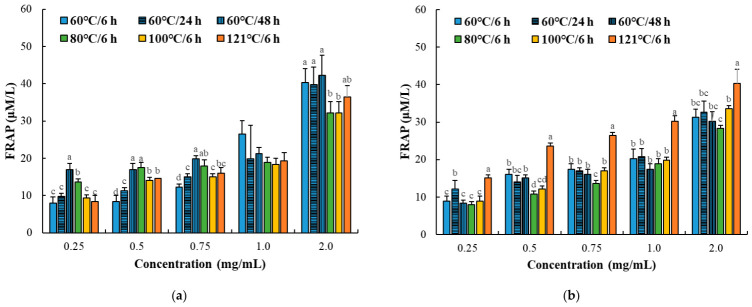
Ferric reducing antioxidant power (FRAP) of myofibrillar (**a**) and sarcoplasmic (**b**) protein fractions (0.25–2.0 mg/mL) from Korean native black goat under different thermal conditions. Error bars represent standard deviation (*n* = 3). a–d: Different letters indicate significant differences among treatments within the same concentration (*p* < 0.05).

FRAP values increased with thermal intensity, particularly in the myofibrillar fraction, although the magnitude of change was less pronounced compared to radical scavenging and metal-chelating activities (Figure 5; Table 2). At a fixed concentration (2 mg/mL), the myofibrillar fraction exhibited higher reducing power under moderate-to-high thermal conditions, whereas the sarcoplasmic fraction showed relatively stable or modest increases. The increase in reducing power reflects enhanced electron-donating capacity, which is associated with both protein degradation and exposure of reactive amino acid residues. Aromatic and sulfur-containing amino acids are known to contribute to reducing activity through electron transfer mechanisms [35]. Previous studies have similarly reported that thermal processing and controlled hydrolysis may improve ferric reducing capacity through exposure of electron-donating residues and formation of low-molecular-weight antioxidant peptides in meat protein systems [28,29].

However, the relatively smaller variation in FRAP compared to radical scavenging and iron-chelating activities suggests that ferric reducing power is less sensitive to protein degradation alone and is more strongly influenced by the overall structural state of the protein system. Unlike DPPH scavenging, which is highly responsive to electron-donating compounds and reactive amino acid residues, FRAP reflects the cumulative reducing potential of the entire soluble protein matrix under acidic conditions. Therefore, structural factors such as protein unfolding, conformational stability, aggregation state, and accessibility of redox-active residues may substantially affect reducing power. Moderate thermal processing may promote exposure of buried electron-donating groups, whereas excessive heating can simultaneously induce aggregation and reduced molecular accessibility, thereby limiting further enhancement of reducing activity despite ongoing protein degradation. This interpretation is consistent with recent studies reporting that thermally induced structural modification does not always proportionally increase ferric reducing capacity in meat protein systems, because aggregation and conformational stabilization may partially offset the beneficial effects of protein degradation and residue exposure [30,36]. Therefore, the present results suggest that ferric reducing activity in thermally processed goat protein systems might be related to a balance between protein degradation, structural accessibility, and aggregation behavior rather than by protein degradation alone.

It should be noted that the present study provides indirect evidence of protein degradation based on α-amino group content and SDS–PAGE patterns, and does not include direct identification of peptide sequences or molecular structural characterization. Nevertheless, distinct changes in antioxidant responses were clearly observed depending on thermal processing conditions and protein fractions, suggesting that heat-induced structural modifications differentially affected the functional properties of myofibrillar and sarcoplasmic protein systems. Therefore, the findings should be interpreted as a preliminary screening of fraction-dependent antioxidant potential rather than definitive mechanistic evidence of bioactive peptide formation.

### 3.3. Cluster Analysis

**Figure 6:**
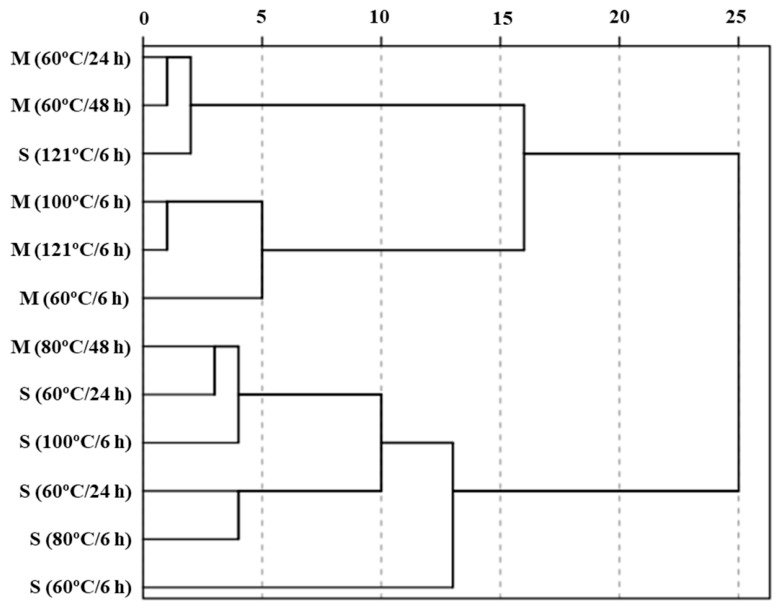
Dendrogram based on antioxidant activity-related variables (DPPH, OH radical scavenging activity, iron-chelating ability, and FRAP) of myofibrillar and sarcoplasmic protein fractions from Korean native black goat under different thermal conditions (60 °C for 6–48 h, 80 °C for 6 h, 100 °C for 6 h, and 121 °C for 6 h). M, myofibrillar protein; S, sarcoplasmic protein.

Hierarchical cluster analysis was performed to classify samples based on their overall antioxidant activity profiles, including DPPH, hydroxyl radical scavenging activity, iron-chelating ability, and FRAP (Figure 6; Table 3). The samples were grouped into four distinct clusters, reflecting differences in antioxidant mechanisms associated with protein fraction and thermal processing conditions.

Cluster 1 consisted of a single sample, S (60 °C/6 h), which was characterized by relatively low radical scavenging activity (higher EC_50_ values) but high reducing power (FRAP). This unique profile suggests that under mild thermal conditions, sarcoplasmic proteins retain reducing capacity without extensive structural modification, resulting in limited radical scavenging but relatively strong electron-donating activity.

Cluster 2 included primarily myofibrillar protein samples subjected to moderate-to-high thermal conditions. This cluster exhibited consistently low EC_50_ values for DPPH and hydroxyl radical scavenging activities, indicating strong antioxidant capacity. The grouping of these samples suggests that myofibrillar proteins are highly responsive to thermal processing, leading to increased structural modification and fragmentation patterns. This is consistent with the observed increase in α-amino group content and SDS–PAGE fragmentation patterns, supporting that structural modification may contribute to the observed antioxidant responses in this cluster.

Cluster 3 comprised samples that showed relatively high EC_50_ values for iron-chelating activity but moderate radical scavenging activity. This indicates that antioxidant activity in this cluster is less associated with metal-chelating mechanisms and more dependent on radical scavenging capacity. The presence of both myofibrillar and sarcoplasmic samples in this cluster suggests intermediate structural modification.

Cluster 4 consisted mainly of sarcoplasmic protein samples subjected to moderate-to-high thermal treatments. These samples were characterized by relatively high EC_50_ values for radical scavenging activity but moderate iron-chelating activity. This clustering pattern suggests that thermal processing of sarcoplasmic proteins primarily induces structural disruption and aggregation rather than extensive fragmentation, resulting in limited improvement in radical scavenging activity.

Overall, the clustering results clearly demonstrate that antioxidant properties are not influenced by a single mechanism but rather by the combined effects of protein structure and thermal processing. It may be associated with structural differences between protein fractions, where fibrous myofibrillar proteins are more susceptible to heat-induced unfolding and fragmentation, whereas globular sarcoplasmic proteins tend to have aggregation during heating. These distinct structural responses may partially explain the fraction-dependent antioxidant patterns observed in the present study. These findings suggest that protein fraction-dependent structural characteristics may influence the extent of thermal-induced functional modification and antioxidant response patterns in Korean native black goat proteins. However, because direct molecular characterization was not performed, the observed antioxidant responses should be interpreted as preliminary screening-level evidence associated with heat-induced structural modification and protein degradation patterns rather than definitive mechanistic evidence of peptide-mediated antioxidant activity.

## 4. Conclusions

This study demonstrated that thermal processing differentially influenced antioxidant response patterns in myofibrillar and sarcoplasmic protein fractions from Korean native black goat. Myofibrillar proteins showed greater responsiveness to thermal treatment, whereas sarcoplasmic proteins exhibited relatively limited changes associated with aggregation-dominant behavior. The results further indicated that antioxidant responses were both fraction-dependent and assay-dependent, suggesting that heat-induced structural modification may influence antioxidant assay behavior differently depending on protein fraction and processing conditions. However, the present study was conducted as a preliminary in vitro screening investigation and did not include direct molecular characterization of peptides or structural analyses. Therefore, the findings should be interpreted as exploratory evidence of fraction-dependent antioxidant responses rather than definitive mechanistic evidence of peptide-mediated antioxidant activity or functional food efficacy. In addition, because gastrointestinal digestion, bioavailability, and in vivo validation were not performed, the present findings have limited direct relevance to functional food efficacy and should be interpreted primarily as preliminary in vitro screening observations. Thus, further studies incorporating peptide profiling, structural characterization, digestion models, and biological validation are necessary to clarify the underlying mechanisms and practical relevance of the observed responses.

## Figures and Tables

**Table 1:** α-Amino group content (mg casein equivalents/mL) of myofibrillar and sarcoplasmic protein fractions from Korean native black goat under different thermal conditions.

Fraction	60 °C/6 h	60 °C/24 h	60 °C/48 h	80 °C/6 h	100 °C/6 h	121 °C/6 h	*p* Value
Myofibrillar protein	0.34 ± 0.07 d	2.39 ± 0.12 c	6.26 ± 0.46 a	4.68 ± 0.07 b	4.80 ± 0.45 b	10.14 ± 2.04 a	<0.001
Sarcoplasmic protein	4.42 ± 0.09 c	4.72 ± 0.13 c	4.90 ± 0.08 c	5.64 ± 0.36 b	5.10 ± 0.58 bc	7.47 ± 0.92 a	<0.001

Values are expressed as mean ± standard deviation (*n* = 3). a–d: Different letters within the same row indicate significant differences among treatments (*p* < 0.05).

**Table 2:** Antioxidant activity of myofibrillar and sarcoplasmic protein fractions from Korean native black goat under different thermal conditions, expressed as EC_50_ values and activity at a fixed concentration.

Protein Type	Extraction Condition (Temperature/Time)	DPPH EC_50_ (mg/mL)	OH EC_50_ (mg/mL)	Iron-Chelating EC_50_ (mg/mL)	FRAP at 2 mg/mL
Myofibrillar protein	60 °C/6 h	1.95 ± 0.09 c	1.77 ± 0.12 a	1.86 ± 0.40 f	40.29 ± 0.378 ab
	60 °C/24 h	1.98 ± 0.10 c	0.95 ± 0.02 cd	3.80 ± 0.47 de	39.80 ± 4.59 ab
	60 °C/48 h	1.96 ± 0.17 c	0.78 ± 0.02 d	4.32 ± 0.42 d	42.19 ± 5.41 a
	80 °C/6 h	1.47 ± 0.03 c	0.91 ± 0.04 cd	3.47 ± 0.08 e	32.19 ± 2.97 bc
	100 °C/6 h	1.55 ± 0.11 c	1.05 ± 0.12 c	0.39 ± 0.01 g	32.10 ± 2.93 bc
	121 °C/6 h	1.49 ± 0.04 c	1.28 ± 0.08 b	0.70 ± 0.01 g	36.48 ± 3.81 abc
Sarcoplasmic protein	60 °C/6 h	3.58 ± 0.20 c	1.92 ± 0.25 a	7.50 ± 1.11 a	32.90 ± 0.73 bc
	60 °C/24 h	6.38 ± 0.49 b	1.30 ± 0.17 b	2.98 ± 0.18 e	32.05 ± 1.27 bc
	60 °C/48 h	10.22 ± 3.39 a	0.85 ± 0.02 d	7.25 ± 0.62 ab	31.90 ± 2.31 bc
	80 °C/6 h	11.52 ± 2.63 a	0.92 ± 0.03 cd	5.64 ± 0.14 c	28.38 ± 0.82 c
	100 °C/6 h	7.34 ± 0.63 b	0.54 ± 0.01 e	3.44 ± 0.30 e	33.62 ± 0.44 abc
	121 °C/6 h	3.19 ± 0.41 c	0.55 ± 0.03 e	6.48 ± 0.48 b	40.29 ± 3.78 ab
Muscle effect (M)		<0.001	0.004	<0.001	NS
Extraction effect (E)		<0.001	<0.001	<0.001	0.001
Interaction		<0.001	<0.001	<0.001	0.022

Values are expressed as mean ± standard deviation (*n* = 3). a–g: Different letters within the same column indicate significant differences among treatments (*p* < 0.05). EC_50_ values represent the concentration required to achieve 50% activity. For FRAP, values are expressed at a fixed concentration (2 mg/mL) due to the inability to determine EC_50_ within the tested concentration range. Lower EC_50_ values indicate higher antioxidant activity.

**Table 3:** Mean z-scores of antioxidant activity parameters for each cluster and corresponding treatments.

Factor	Cluster 1 (*n* = 1)	Cluster 2 (*n* = 5)	Cluster 3 (*n* = 3)	Cluster 4 (*n* = 3)
Z-score (DPPH radical EC_50_)	−0.225	−0.506	−0.558	1.476
Z-score (OH radical EC_50_)	1.480	−0.887	0.371	0.615
Z-score (Iron chelating EC_50_)	−0.765	−0.197	1.201	−0.619
Z-score (FRAP)	1.972	0.446	−0.708	−0.693
Assigned treatment	S (60 °C/6 h)	M (60 °C/6 h),	M (60 °C/24 h),	S (60 °C/48 h),
		M (80 °C/6 h),	M (60 °C/48 h),	S (80 °C/6 h),
		M (100 °C/6 h),	S (121 °C/6 h)	S (100 °C/6 h)
		M (121 °C/6 h),		
		S (60 °C/24 h)		

Values represent mean z-scores within each cluster. Z-scores were calculated after standardization of antioxidant activity parameters (DPPH radical scavenging activity, hydroxyl radical scavenging activity (OH), iron-chelating ability, and ferric reducing antioxidant power (FRAP)). For EC_50_ values, lower z-scores indicate higher antioxidant activity, whereas for FRAP, higher z-scores indicate greater reducing power. M, myofibrillar fraction; S, sarcoplasmic fraction.

## Data Availability

The original contributions presented in this study are included in the article. Further inquiries can be directed to the corresponding author.
